# Adhesion of Pancreatic Cancer Cells in a Liver-Microvasculature Mimicking Coculture Correlates with Their Propensity to Form Liver-Specific Metastasis *In Vivo*

**DOI:** 10.1155/2014/241571

**Published:** 2014-05-11

**Authors:** Mohammad Mahfuz Chowdhury, Mathieu Danoy, Farhana Rahman, Marie Shinohara, Shohei Kaneda, Kiyotaka Shiba, Naoya Fujita, Teruo Fujii, Yasuyuki Sakai

**Affiliations:** ^1^Institute of Industrial Science, The University of Tokyo, 4-6-1 Komaba, Meguro-ku, Tokyo 153-8505, Japan; ^2^LIMMS/CNRS UMI2820 Institute of Industrial Science, The University of Tokyo, 4-6-1 Komaba, Meguro-ku, Tokyo 153-8505, Japan; ^3^Cancer Institute, Japanese Foundation for Cancer Research, 3-8-31 Ariake, Koto-ku, Tokyo 135-8550, Japan; ^4^The Cancer Chemotherapy Center, Japanese Foundation for Cancer Research, 3-8-31 Ariake, Koto-ku, Tokyo 135-8550, Japan

## Abstract

Organ-specific characteristic of endothelial cells (ECs) is crucial for specific adhesion of cancer cells to ECs, which is a key factor in the formation of organ-specific metastasis. In this study, we developed a coculture of TMNK-1 (immortalized liver sinusoidal ECs) with 10T1/2 (resembling hepatic stellate cells) to augment organ-specific characteristic of TMNK-1 and investigated adhesion of two pancreatic cancer cells (MIA-PaCa-2 and BxPC-3) in the culture. MIA-PaCa-2 and BxPC-3 adhesion in TMNK-1+10T1/ 2|coating culture (TMNK-1 monolayer over 10T1/2 layer on collagen coated surface) were similar. However, in TMNK-1+10T1/ 2|gel (coculture on collagen gel surface), MIA-PaCa-2 adhesion was significantly higher than BxPC-3, which was congruent with the reported higher propensity of MIA-PaCa-2 than BxPC-3 to form liver metastasis *in vivo*. Notably, as compared to BxPC-3, MIA-PaCa-2 adhesion was lower and similar in TMNK-1 only culture on the collagen coated and gel surfaces, respectively. Investigation of the adhesion in the representative human umbilical vein ECs (HUVECs) cultures and upon blocking of surface molecules of ECs revealed that MIA-PaCa-2 adhesion was strongly dependent on the organ-specific upregulated characteristics of TMNK-1 in TMNK-1+10T1/ 2|gel culture. Therefore, the developed coculture would be a potential assay for screening novel drugs to inhibit the liver-microvasculature specific adhesion of cancer cells.

## 1. Introduction

Metastasis is a series of well-defined interrelated steps through which cancer/tumor cells of a primary tumor in an organ form secondary tumors/metastases in other organs. During metastasis process cancer cells from a primary tumor locally invade the surrounding tissue, enter the nearby vascular system (intravasation), and go into the vascular system of a distant organ mainly by blood circulation. Then, they arrest and adhere to the vascular wall (i.e., endothelium) of the organ and migrate inside the organ parenchyma (extravasation), survive in the microenvironment in ways that facilitate their proliferation, and finally form macroscopic secondary tumors (colonization) [1]. Secondary tumors but not the primary tumors are responsible for 90% of cancer-associated mortality [1]. Therefore, a comprehensive understanding of the mechanism involved in each step of metastasis is necessary to develop novel drugs to reduce the mortality of cancer patients [2].

Theoretically, circulating cancer/tumor cells (CTCs) can disseminate and form secondary tumors in all organs [3]. However, clinical observations indicate that cancer cells show a remarkable preference for certain organs to form secondary tumors [4, 5]. For example, cancer cells originating from breast frequently metastasize to bone, liver, brain, and lung, whereas cancer cells originating in pancreas preferentially metastasize to liver and lung [4, 5]. In addition, cancer cell lines originating from the same organ can form metastases in a particular organ at different rates. For example, orthotopic implantation of pancreatic cancer cell MIA-PaCa-2 in the mouse pancreas caused liver metastasis at a higher rate (almost five times higher) than another pancreatic cell line BxPC-3 [6].

Organ-specific arrest and adhesion of cancer cells to the vascular system of the organs are an initiating and a crucial step responsible for the organ-specific pattern of metastasis. [3, 4, 7–9]. Cancer cell can be specifically arrested in an organ vasculature depending on the various mechanical characteristics (anatomical, size restriction, and blood flow) of the vascular system [4]. Moreover, cancer cell can preferentially adhere to the endothelium/endothelial cells (ECs) of an organ owing to the specific and efficient adhesive interactions between cancer cell and endothelium [8, 9].

Among the numerous factors present in vascular environment, characteristic/phenotype of ECs is one of the crucial factors that influence the adhesive interactions between CTCs and ECs [8, 10]. Although ECs share certain common properties, they show variation in regard to structure, antigenic and cell surface determinants, adhesion molecules, and metabolic functions from organ to organ [11]. The unique organ-specific phenotype of the ECs is undoubted determinant for the specific adhesion of cancer cells and thereby plays a crucial role in the formation of organ-specific pattern of metastasis [8, 10].

Microvascular wall is composed of two interacting cell types. ECs form the inner lining of the wall and perivascular cells, referred to as pericytes (PCs), and envelop the surface of the vascular tube [12]. Pericytes form a network of long cytoplasmic processes which directly contact ECs and therefore communicate with ECs by direct physical contact and paracrine signaling pathways [12]. Communication between ECs and PCs is a crucial component of the vascular microenvironment which modulates ECs as well as PCs phenotype [12–16].

After the lymph nodes, liver is the second most commonly affected organ by metastasis [17]. Liver involvement is a major determinant of survival from cancer as 30–70% of patients dying of cancer have hepatic metastases [17].* In vivo* studies suggest that successful arrest/adhesion of cancer cells to the liver is not merely a mechanical process but depends on specific interactions between the cancer cells and the liver endothelium [18, 19]. These interactions contribute to the metastasis efficacy and therefore represent useful therapeutic targets for controlling metastasis process [3, 20].

In this context, the objective of this study is to develop a culture model for liver endothelium, which would preserve liver-specific characteristic of ECs and provide a readily applicable yet improved* in vitro* model to investigate liver-specific adhesion and subsequent processes (e.g., migration) of cancer cells. Culture system of this kind has the potential of screening novel drugs to inhibit interactions between cancer cells and liver endothelium.

In the study, to mimic liver endothelium, we developed a coculture of TMNK-1 (immortalized human liver sinusoidal ECs) [21] and 10T1/2 (model cells for PCs which are equivalent to hepatic stellate cells) [12, 22] under various conditions. We investigated the influence of TMNK-1 characteristics in the cultures on the adhesion behavior of two pancreatic cancer cell lines (MIA-PaCa-2 and BxPC-3) and correlated the outcome with their propensity to form metastasis in the liver [6]. In addition, we confirmed the correlation by examining adhesion of these cancer cells in HUVECs (replacing TMNK-1) containing cultures and evaluating the role of liver-microvasculature specific surface molecules on adhesion of the cancer cells.

## 2. Materials and Methods

### 2.1. Routine Cultures of 10T1/2, TMNK-1, HUVECs, MIA-PaCa-2, and BxPC-3

10T1/2, HUVECs, MIA-PaCa-2, and BxPC-3 were purchased from Japan Collection of Research Bioresources Cell Bank, Lonza, Inc., AntiCancer, Inc., and DS Pharma, Japan. TMNK-1 cells were kindly provided by Dr. Naoya Kobayashi, Okayama University, Japan, respectively. These cells were routinely cultured in 100 mm tissue culture treated polystyrene (TCPS) dishes. Cell inoculation densities were 1 × 10^5^ cells/dish (for 10T1/2 and TMNK-1), 2 × 10^5^ cells/dish for HUVECs, and 1 × 10^6^ cells/dish (for MIA-PaCa-2 and BxPC-3). All cells were cultured for 4 days before the subsequent subculture. Culture medium was changed once during the culture period. Composition of the culture medium for 10T1/2, TMNK-1, MIA-PaCa-2, and BxPC-3 was high glucose DMEM (DMEM; Gibco) containing 10% fetal bovine serum (FBS; Gibco), 1% MEM nonessential amino acids (Gibco), 100 U/mL penicillin, and 100 U/mL streptomycin (Gibco). Culture medium for HUVECs was EGM-2 BulletKit (Lonza, Inc.). The cells were maintained in a 37°C humidified environment containing 5% CO_2_.

### 2.2. Experimental Culture of TMNK-1 (or HUVECs) and TMNK-1 (or HUVECs) + 10T1/2

Diluted (0.15 mg/mL for collagen coating) or concentrated (2.4 mg/mL for collagen gel) solution of collagen 1-P (Nitta Gelatin, Inc., Japan) was prepared according to the manufacturer's instructions. Sufficient volume of diluted or concentrated solution was added to the TCPS well plates (250 *μ*L and 50 *μ*L for each well in the 24-well and 96-well plate, resp.) to cover the whole culture surface. Afterwards, plates were incubated in the laminar flow hood at room temperature (for collagen coating) or in the incubator at 37°C (for collagen gel) for 30 minutes. Plates containing collagen coating solution were aspirated and air-dried in the laminar flow hood for 2 hrs before using them as collagen coated plates, whereas the plates containing concentrated solution were directly used for subsequent inoculation of 10T1/2 cells.

10T1/2 was inoculated at 2 × 10^4^ cells/cm^2^ in the collagen coated TCPS well and on the collagen gel (day 0). On the following day (day 1), TMNK-1 was inoculated at 1.5 × 10^5^ cells/cm^2^ on the 10T1/2 layer in the collagen coated and gel cultures. TMNK-1 alone was also inoculated in the collagen coated TCPS well and on the collagen gel. In a similar fashion, we prepared culture of HUVECs with or without 10T1/2 on collagen gel surface. Notably, the ratio of 10T1/2 density to TMNK-1 (or HUVECs) density was kept similar to the ratio of hepatic stellate cells to human liver sinusoidal endothelial cells (hLSECs) in the liver [23]. Culture medium of the wells was changed every day and the wells were used for cancer cell adhesion assay on day 4. In some experiments, 10T1/2 and TMNK-1 were stained with PKH67 and PKH26 dyes (Sigma Aldrich) according to the manufacturer's instructions for visualizing the morphology of the cells. From here on, TMNK-1 only and TMNK-1 + 10T1/2 culture in the collagen coated wells would be designated as TMNK-1|coating and TMNK-1 + 10T1/2|coating culture, respectively, whereas respective cultures in the gel would be designated as TMNK-1 (of HUVECs)|gel and TMNK-1 (or HUVECs) + 10T1/2|gel.

### 2.3. Adhesion Assay of MIA-PaCa-2 and BxPC-3 in the Experimental Culture

Both cancer cells were stained with a cytoplasmic dye (CMFDA, Life Technologies, Inc.) according to the manufacturer's instruction. Then, the cancer cells were suspended in a culture medium containing 0.5% FBS; MIA-PaCa-2 or BxPC-3 cells were added at 5 × 10^4^ cells/well in the experimental cultures in 96-well plate and incubated for 90 minutes in the incubator. Afterwards, the unattached cancer cells were gently washed with the culture medium three times and 200 *μ*L of cell lysis buffer was added in each well (0.5% Triton X-100 solution in PBS, Sigma-Aldrich) to release the dye from cancer cells into the buffer.

To measure the dye intensity in the buffer, 100 *μ*L of the buffer was transferred from each well to a well in a glass-bottom 96-well plate (Iwaki). The intensity was measured by using a fluorometric plate reader (PerkinElmer) at 485 nm excitation and 535 nm emission. For each culture condition, dye intensity in the buffer transferred from the respective culture without any cancer cells was used as blank. The number of adhered cancer cells in the cultures was calculated from the calibration curves prepared by measuring intensity in the buffer which was prepared by lysing stained cancer cells at different concentrations.

### 2.4. Immunostaining

In case of TMNK-1|coating or TMNK-1 + 10T1/2|coating culture, respective cultures in glass bottom 96-well plate were used for immunostaining of various markers in TMNK-1. However, owing to the thickness of the gel culture, which can impair confocal microscopy, we prepared vertical cross sections of the gel cultures embedded in paraffin for the immunostaining (Saipaso Research Center, Tokyo, Japan). We also prepared vertical cross sections stained with haematoxylin and eosin (HE) and took images of these samples with a transmitted light microscope (Olympus, Inc.).

The primary antibodies against the markers were anti-VAP-1 (2 *μ*g/mL, Lifespan Biosciences), anti-LYVE-1 (2 *μ*g/mL, Reliatech GmbH), anti-Stabilin-1 (2 *μ*g/mL, Santa Cruz, Inc.), and anti-ICAM-1 (2.5 *μ*g/mL, R&D, Inc.). The secondary antibody was conjugated with Alexa Fluor 488 or 594 (5 *μ*g/mL, Life Technologies, Inc.). Immunostaining of the cultures and the vertical cross sections was carried out according to the protocols provided by manufacturers or described elsewhere [24].

Images of the samples were taken by a confocal microscope (Olympus, Inc.). The average pixel intensity of the whole image area (for immunostaining of the collagen coated cultures) and of a line which coincided with the TMNK-1 layer (topmost layer) in the vertical cross sections of the gel cultures was measured by using ImageJ software (NIH). To compare the image intensities on a common basis, intensity of the TMNK-1 + 10T1/2|coating and TMNK-1 + 10T1/2|gel cultures was normalized by the intensity of the TMNK-1|coating and TMNK-1|gel culture, respectively. At least five images from two samples for each culture condition were used to measure the intensity.

### 2.5. Adhesion of MIA-PaCa-2 and BxPC-3 under Blocking of Surface Molecules of ECs in the Cultures

To block ICAM-1, experimental wells were incubated with anti-ICAM-1 antibody (10 *μ*g/mL, R&D) or isotype IgG (as control, 10 *μ*g/mL, R&D) for three hours before the addition of cancer cells in the wells. In the VAP-1 blocking experiments, before the addition of cancer cells, the wells were incubated for 30 minutes with a peptide GGGGGGGGK (as control peptide; termed as P1 hereafter) or GGGGKGGGG (effective peptide; termed as P2 hereafter). Though both of these peptides fit in the active-site channel of VAP-1, P2 inhibits VAP-1 more efficiently than P1 as P1-VAP-1 contacts are much poorer than P2-VAP-1 contacts [25]. Synthesized peptides were obtained from Life Technologies, Inc.; and working concentration of both peptides was 0.4 mg/mL.

### 2.6. Statistical Analysis

Student's* t*-test for comparing two groups was performed for statistical evaluation by using the demo version of GraphPad software (GraphPad Software, Inc.). Differences with *P* < 0.05 (*), *P* < 0.01 (**), or *P* < 0.001 (***) were considered to be statistically significant. All data were presented as the mean ± SEM (or −SD).

## 3. Results

### 3.1. TMNK-1 + 10T1/2 Culture in the Collagen Coated Well (TMNK-1 + 10T1/2|Coating)

**Figure 1:**
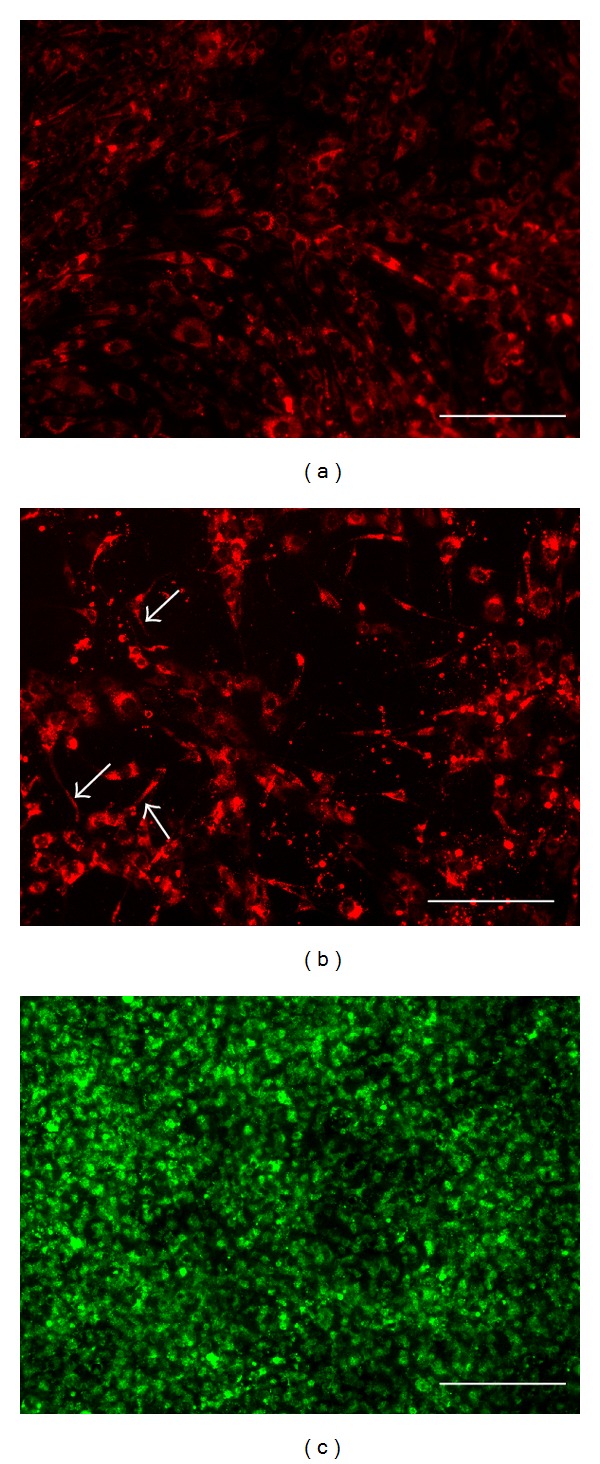
Images of the cells in the collagen coated well cultures on day 4. (a) Monolayer morphology of 10T1/2 cells (red) without TMNK-1. (b) 10T1/2 cells (red) show a dispersed morphology containing cytoplasmic processes (indicated by arrows) with TMNK-1. (c) Monolayer of TMNK-1 (green) over 10T1/2 cells. Scale bar represents 200 *μ*m.

To achieve close contact between 10T1/2 and TMNK-1, which is crucial for the communication between these cell types [12, 26, 27], we first inoculated 10T1/2 in the well. Then, on the following day, we inoculated TMNK-1 which subsequently formed continuous monolayer over the 10T1/2 layer (Figure 1). Interestingly, upon the addition of TMNK-1, monolayer morphology of 10T1/2 changed into a dispersed morphology containing cytoplasmic processes (Figure 1) which is a hallmark of endothelial cell and pericyte interactions [22].

### 3.2. MIA-PaCa-2 and BxPC-3 Adhesion in the TMNK-1 + 10T1/2|Coating Culture

**Figure 2:**
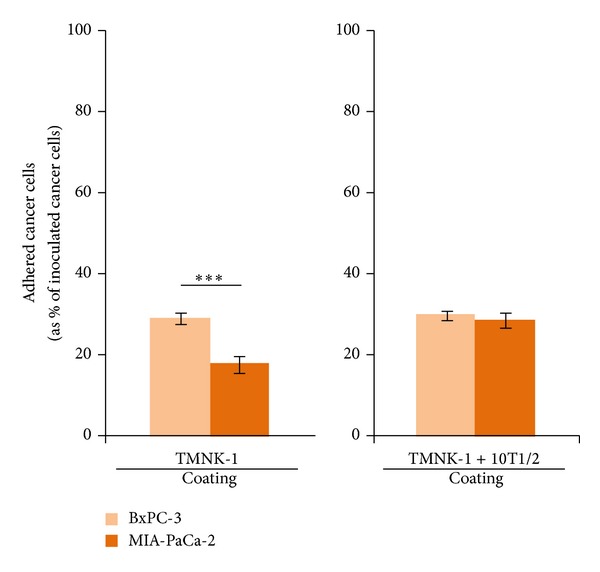
BxPC-3 and MIA-PaCa-2 adhesion in the TMNK-1 only and TMNK-1 + 10T1/2 cultures in the collagen coated wells. Columns and error bars represent mean ± SEM of two independent experiments. Each independent experiment had six wells for each culture condition. Statistical significance is shown using symbols *(*P* < 0.05), **(*P* < 0.01), and ***(*P* < 0.001).

To study the effect of TMNK-1 characteristic on the adhesion behavior of cancer cells, we investigated MIA-PaCa-2 and BxPC-3 adhesion in the TMNK-1 + 10T1/2|coating and TMNK-1|coating (as a basis for comparison) cultures. In fact, cancer cells adhesion is a common assay to characterize their propensity to form metastases in a certain organ* in vitro* [28–30]. As compared to BxPC-3, MIA-PaCa-2 adhesion was lower and similar in the TMNK-1|coating and TMNK-1 + 10T1/2|coating culture, respectively (Figure 2). However, MIA-PaCa-2 adhesion in both cultures was not congruent with the reported higher propensity of MIA-PaCa-2 than BxPC-3 to form liver metastasis* in vivo* (almost five times higher) [6].

### 3.3. TMNK-1 + 10T1/2 Culture on Collagen Gel Surface (TMNK-1 + 10T1/2|Gel)

Rigid collagen coated TCPS might not be an innate substrate for the culture, thereby impairing the characteristics of the TMNK-1 + 10T1/2 culture, which in turn affected the cancer cell adhesion. We speculated that collagen gel might provide a better substrate (as gel bears a better resemblance to the soft nature of an organ) which would enable an innate adhesion of cancer cells. To examine that, we cultured TMNK-1 with 10T1/2 on the top of collagen gel instead of the collagen coated TCPS. We also cultured TMNK-1 alone on the gel surface (TMNK-1|gel, as a basis for comparison).

**Figure 3:**
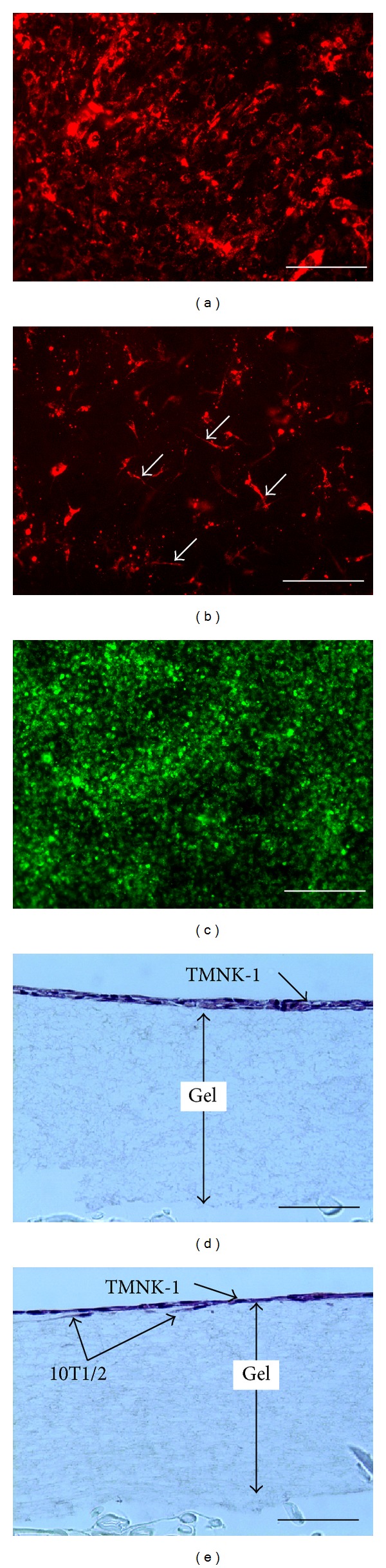
Images of the cells in the collagen gel cultures on day 4. (a) Monolayer morphology of 10T1/2 cells (red) without TMNK-1. (b) 10T1/2 cells (red) show a dispersed morphology containing cytoplasmic processes (indicated by arrows) with TMNK-1. (c) TMNK-1 cells (green) form monolayer over 10T1/2. (d) Haematoxylin and eosin (HE) staining of vertical cross section of TMNK-1 only culture. (e) HE staining of TMNK-1 + 10T1/2 culture. Some 10T1/2 cells in the culture can be distinguished by their elongated morphology beneath the TMNK-1 layer. Scale bars represent 200 *μ*m (for (a), (b), and (c)) and 100 *μ*m (for (d) and (e)).

Similar to the collagen coated TCPS culture, monolayer morphology of 10T1/2 on the gel changed into a network morphology containing cytoplasmic processes upon the inoculation of TMNK-1 (Figures 3(a) and 3(b)). However, as compared to the TMNK-1 + 10T1/2|coating culture, 10T1/2 in the TMNK-1 + 10T1/2|gel culture showed lower coverage and more cytoplasmic processes (Figures 1(b) and 3(b)). TMNK-1 formed continuous monolayer over the 10T1/2 layer (Figure 3(c)). HE staining of the vertical cross sections of the gel cultures indicated no extensive growth or migration of TMNK-1 or 10T1/2 in the gel cultures even after 5 days of culture (Figures 3(d) and 3(e)).

### 3.4. MIA-PaCa-2 and BxPC-3 Adhesion in the TMNK-1 + 10T1/2|Gel Culture

**Figure 4:**
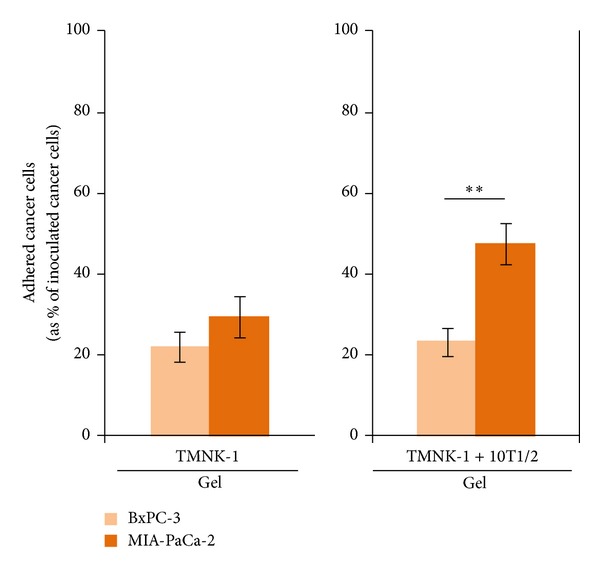
BxPC-3 and MIA-PaCa-2 adhesion in the TMNK-1 only and TMNK-1 + 10T1/2 in the collagen gel cultures. Columns and error bars represent mean ± SEM of two independent experiments. Each independent experiment had six wells for each culture condition. Statistical significance is shown using symbols *(*P* < 0.05), **(*P* < 0.01), and ***(*P* < 0.001).

We investigated the cancer cell adhesion in the TMNK-1 + 10T1/2|gel and TMNK-1|gel cultures (as a basis for comparison). In the TMNK-1|gel, MIA-PaCa-2 adhesion showed a higher tendency than BxPC-3 (though not statistically significant, Figure 4). However, MIA-PaCa-2 adhesion was significantly higher than BxPC-3 in TMNK-1 + 10T1/2|gel (Figure 4). The higher MIA-PaCa-2 adhesion in the TMNK-1 + 10T1/2|gel culture was congruent with its higher propensity than BxPC-3 to form liver metastasis* in vivo* [6].

### 3.5. Characteristics of TMNK-1 in Various Culture Conditions

The observed variation in cancer cell adhesion in the cultures might have resulted from the different characteristics of TMNK-1 under various culture conditions (rigid TCPS, gel, and 10T1/2). To confirm this, we investigated TMNK-1 characteristics in the different cultures by analyzing the expression of various makers (VAP-1, LYVE-1, Stabilin-1, and ICAM-1) which are normal phenotypic markers of primary human liver sinusoidal endothelial cells (hLSECs) [31–33].

**Figure 5:**
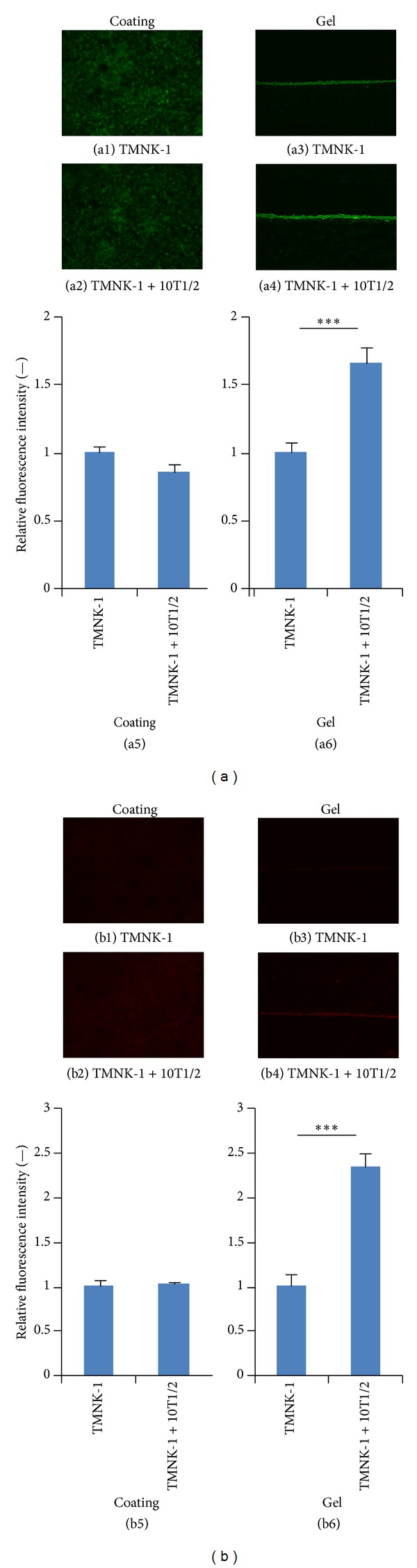
Expression of TMNK-1 phenotypic marker VAP-1 (a) and LYVE-1 (b) in various culture conditions. (a1), (a2), (b1), and (b2) and (a3), (a4), (b3), and (b4) show representative images showing the expression of the respective markers in the collagen coated and gel cultures, respectively. Similarly, (a5) and (b5) and (a6) and (b6) show the average pixel intensity of the immunostaining in the cultures. Intensity was averaged from at least five images taken from two samples for each condition. Columns and error bars represent mean ± SEM. Statistical significance is shown using symbols *(*P* < 0.05), **(*P* < 0.01), and ***(*P* < 0.001).

**Figure 6:**
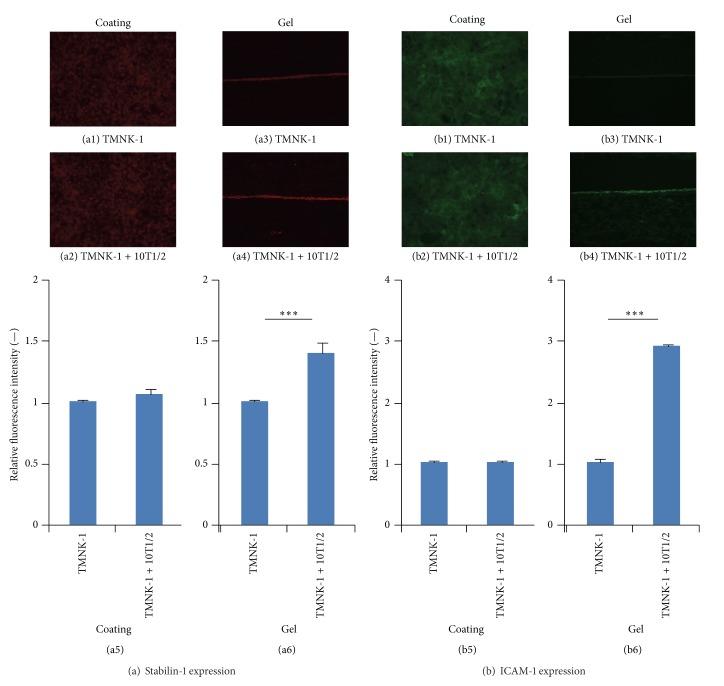
Expression of TMNK-1 phenotypic marker Stabilin-1 (a) and ICAM-1 (b) in various culture conditions. (a1), (a2), (b1), and (b2) and (a3), (a4), (b3), and (b4) show representative images showing the expression of the respective markers in the collagen coated and gel cultures, respectively. Similarly, (a5) and (b5) and (a6) and (b6) show the average pixel intensity of the immunostaining in the cultures. Intensity was averaged from at least five images taken from two samples for each condition. Columns and error bars represent mean ± SEM. Statistical significance is shown using symbols *(*P* < 0.05), **(*P* < 0.01), and ***(*P* < 0.001).

VAP-1, LYVE-1, and Stabilin-1 are newer surface molecule makers to characterize the normal phenotype of hLSECs. They all have important role on leukocyte adhesion to endothelium. Interestingly, all these phenotypic markers were significantly upregulated in TMNK-1 in the TMNK-1 + 10T1/2|gel culture (Figures 5 and 6(a)). ICAM-1 is constitutively expressed highly in hLSECs and plays an important role in the interactions with cancer cells [10, 33]. Similar to the other markers, ICAM-1 expression was highest in the TMNK-1 + 10T1/2|gel culture (Figure 6(b)). These results indicated that TMNK-1 characteristic was better preserved in the TMNK-1 + 10T1/2|gel, whereas the phenotype in all other cultures was at basal level. These results together with adhesion results confirmed that adhesion of cancer cells strongly depended on the TMNK-1 characteristics in the cultures. More importantly, only in the TMNK-1 + 10T1/2|gel culture, where TMNK-1 preserved upregulated characteristic, MIA-PaCa-2 and BxPC-3 adhesion was congruent with their propensity to form liver metastasis* in vivo*. This is in accord with the crucial role of organ-specific characteristics of ECs on the formation of organ-specific pattern of metastasis.

### 3.6. MIA-PaCa-2 and BxPC-3 Adhesion in HUVECs + 10T1/2|Gel Culture

To confirm that the observed adhesion pattern of cancer cells was only specific to hepatic sinusoidal endothelial cells (i.e., TMNK-1), we investigated cancer cell adhesion in cultures containing ECs from a tissue other than liver. For this, we investigated MIA-PaCa-2 and BxPC-3 adhesion in HUVECs containing cultures: HUVECs + 10T1/2|gel and HUVECs|gel cultures (as a basis for comparison). We opted for gel condition as it provided better microenvironment for the culture as compared to the coating condition. HUVECs formed continuous monolayer on collagen gel with or without 10T1/2 cells (see Supplementary Figure 1 in the Supplementary Material available online at http://dx.doi.org/10.1155/2014/241571).

**Figure 7:**
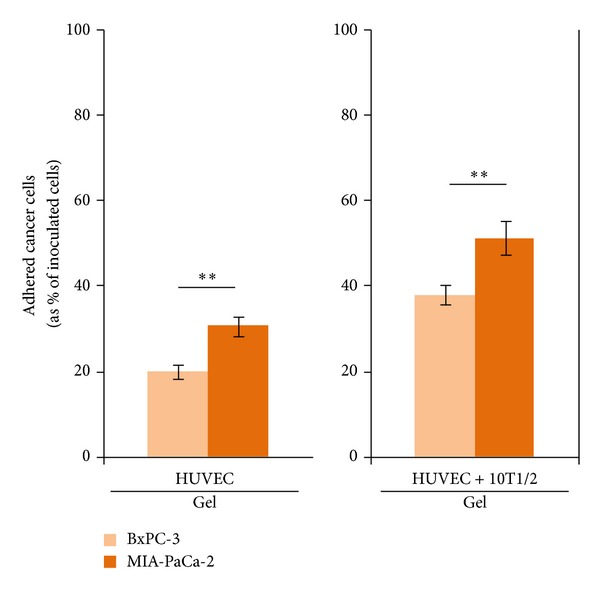
BxPC-3 and MIA-PaCa-2 adhesion in the HUVECs only and HUVECs + 10T1/2 in the collagen gel cultures. Columns and error bars represent mean ± SEM of two independent experiments. Each independent experiment had six wells for each culture condition. Statistical significance is shown using symbols *(*P* < 0.05), **(*P* < 0.01), and ***(*P* < 0.001).

Adhesion of MIA-PaCa-2 was significantly higher than BxPC-3 in both HUVECs|gel and HUVECs + 10T1/2|gel cultures (Figure 7). In addition, there was a significant increase in adhesion of both cancer cells in HUVECs + 10T1/2|gel culture as compared to that in HUVECs|gel culture (Figure 7). However, in case of TMNK-1 containing gel cultures, similar significant increase was observed only in the adhesion of MIA-PaCa-2 (Figure 4). These observations indicated that adhesion capability of MIA-PaCa-2 was inherently higher than BxPC-3 and ECs from different tissue locations modulated adhesion of these cancer cells in a different way.

### 3.7. Adhesion of MIA-PaCa-2 and BxPC-3 in the Cultures under Blocking Condition

Higher adhesion of MIA-PaCa-2 than BxPC-3 in both TMNK-1 and HUVECs containing cultures undermined the correlation between adhesion of these cancer cells and their propensity to form organ-specific metastasis. However, from the observation that cancer cell adhesion was modulated differently in different EC cultures, we speculated that some liver-microvasculature specific surface molecules of ECs modulated adhesion of cancer cells in the TMNK-1 but not HUVECs containing cultures.

To confirm this, we investigated cancer cell adhesion in the cultures by blocking two surface molecules of ECs: VAP-1 and ICAM-1. We selected to block VAP-1 and ICAM-1 on the following two reasons. Firstly, our preliminary blocking experiments by using specific antibodies indicated that VAP-1 and ICAM-1 influenced MIA-PaCa-2 adhesion in TMNK-1 + 10T1/2|gel culture in a greater extent than others (LYVE-1 and Stabilin-1) (data not shown). Secondly, VAP-1 and ICAM-1 are phenotypic markers of TMNK-1 [31–33] and their amounts in inactivated HUVECs are low [34, 35].

**Figure 8:**
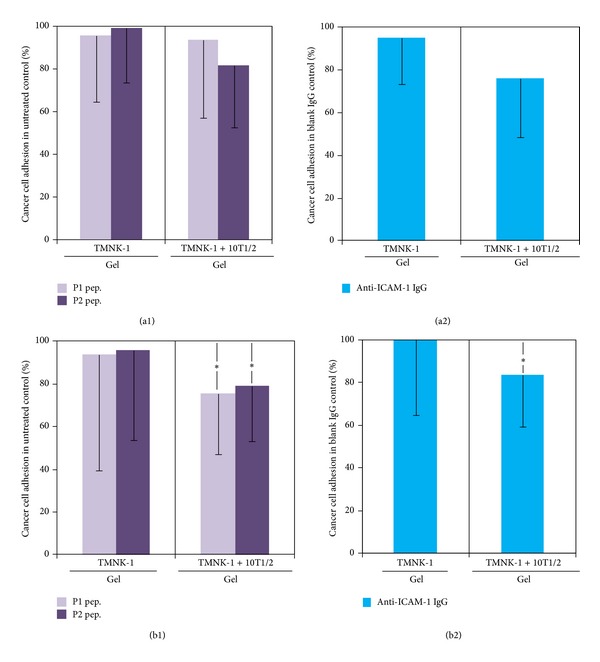
Adhesion of BxPC-3 (a) and MIA-PaCa-2 (b) in the TMNK-1 only and TMNK-1 + 10T1/2 in the collagen gel cultures upon blocking of VAP-1 ((a1) and (b1)) and ICAM-1 ((a2) and (b2)). In the VAP-1 blocking experiment, two peptides were used: GGGGGGGGK (P1, as control) and GGGGKGGGG (P2, effective peptide), which bound poorly and efficiently with VAP-1, respectively [25]. ICAM-1 was blocked with anti-ICAM-1 antibody, while control was an isotype IgG antibody. Columns and error bars represent mean − SD of two to three independent experiments. Each independent experiment had four to six wells for each culture condition. Statistical significance is shown using symbols *(*P* < 0.05), **(*P* < 0.01), and ***(*P* < 0.001).

We first investigated role of VAP-1 and ICAM-1 on the adhesion of cancer cells in the TMNK-1 containing cultures. We inhibited/blocked VAP-1 by using two peptides: P1 (as control peptide) and P2 (as effective peptide). P1 and P2 bind with VAP-1 poorly and efficiently, respectively [25]. We blocked ICAM-1 by using an anti-ICAM-1 antibody. Upon blocking with the peptides or antibody, adhesion of both cancer cells was unchanged in TMNK-1|gel cultures (Figure 8). However, in the TMNK-1 + 10T1/2|gel culture, adhesion of both cancer cells showed a decreasing tendency and MIA-PaCa-2 adhesion decreased significantly under blocking with the peptides or antibody (Figure 8).

**Figure 9:**
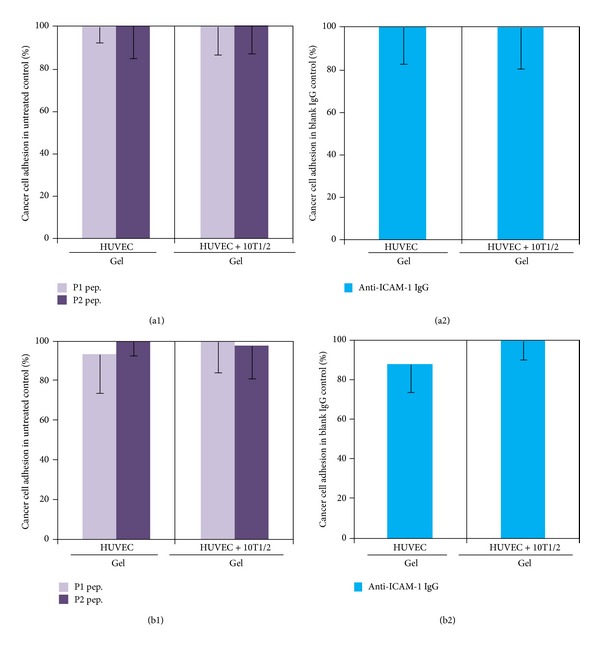
Adhesion of BxPC-3 (a) and MIA-PaCa-2 (b) in the HUVECs only and HUVECs + 10T1/2 in the collagen gel cultures upon blocking of VAP-1 ((a1) and (b1)) and ICAM-1 ((a2) and (b2)). In the VAP-1 blocking experiment, two peptides were used: GGGGGGGGK (P1, as control) and GGGGKGGGG (P2, effective peptide), which bound poorly and efficiently with VAP-1, respectively [25]. ICAM-1 was blocked with anti-ICAM-1 antibody, while control was an isotype IgG antibody. Columns and error bars represent mean − SD of two to three independent experiments. Each independent experiment had four to six wells for each culture condition. Statistical significance is shown using symbols *(*P* < 0.05), **(*P* < 0.01), and ***(*P* < 0.001).

Then, we evaluated the role of VAP-1 and ICAM-1 on the adhesion of cancer cells in HUVECs containing cultures. However, in the respective HUVECs containing cultures, blocking of VAP-1 or ICAM-1 did not cause any change in the adhesion of both cancer cells (Figure 9).

These results indicated that though adhesion capability of MIA-PaCa-2 might be inherently higher than BxPC-3, the higher adhesion of MIA-PaCa-2 in TMNK-1 + 10T1/2|gel culture was controlled by liver-microvasculature specific surface molecules of ECs. Therefore, the higher adhesion of MIA-PaCa-2 than BxPC-3 in the TMNK-1 + 10T1/2|gel, irrespective of the higher adhesion of MIA-PaCa-2 than BxPC-3 in the HUVEC cultures, reflected their propensity to form liver metastasis* in vivo*.

## 4. Discussion

Various studies showed that adhesion of cancer cells in primary culture of organ-specific ECs, ECs cultured on organ-specific matrix components, and cryostat section of organs correlated well with the propensity of cancer cell to form metastasis in certain organs [28–30]. In our study, for the first time, we correlated higher propensity of MIA-PaCa-2 than BxPC-3 to form liver metastasis based on their adhesion behavior in an* in vitro* culture by using continuous cell lines TMNK-1 and 10T1/2 as model cells for organ-specific ECs and PCs.

As we systemically varied culture condition, MIA-PaCa-2 adhesion showed a gradual increase over the BxPC-3 adhesion (by examining Figures 2 and 4 side by side from left to right). This relative change in the MIA-PaCa-2 adhesion compared to the BxPC-3 adhesion, together with the expression of various markers in cultures (Figures 5 and 6), presumably indicated the sequential regulation of TMNK-1 phenotype from basal to elevated level (though some change was not measurable) in the cultures. Therefore, the basal level of TMNK-1 might not have achieved the organ-specific EC characteristic. On the other hand, TMNK-1 + 10T1/2|gel culture achieved the organ-specific EC characteristic, and thereby adhesion of the cancer cells in that culture correlated with their propensity to form liver metastasis.

Although the presence of 10T1/2 did not have any remarkable contribution to the TMNK-1 phenotype in the collagen coated culture, it influenced the phenotype significantly in the gel culture. Two reasons plausibly explained the observation: (1) gel provided more natural and soft substrate for both TMNK-1 and 10T1/2 for establishing effective cellular communications as compared to rigid TCPS surface and (2) substrate stiffness modulated 10T1/2 behavior [36]. Difference in the 10T1/2 morphology between the TMNK-1 + 10T1/2|coating and TMNK-1 + 10T1/2|gel cultures indicated a possible contribution of the gel to the resulting culture microenvironment (Figures 1(b) and 3(b)).

We observed that both less efficient inhibitor (P1; selected to be used as control peptide) and efficient inhibitor (P2) of VAP-1 influenced adhesion of cancer cells (Figure 8). Both of these peptides fit in the active-site channel of VAP-1; however, P1-VAP-1 contacts are much poorer than P2-VAP-1 contacts [25]. Therefore, loose conformation of P1 in the active-site channel of VAP-1 might have been sufficient to inhibit cancer cell adhesion. In addition, Yegutkin and coworkers showed that though P2 inhibited leukocyte adhesion on endothelial cells in a flow-based adhesion assay, P1 did not have any influence on the adhesion [25]. However, we investigated cancer cell adhesion in static condition. Therefore, flow might have some effects on the efficacy of the peptides. This suggests that it is important to screen peptides or other blocking molecules for their efficacy in flow-based cultures besides static cultures.

Among TMNK-1|gel, TMNK-1 + 10T1/2|gel, HUVECs|gel, and HUVECs + 10T1/2|gel, only in the TMNK-1 + 10T1/2|gel adhesion of cancer cells decreased upon blocking of VAP-1 and ICAM-1 (Figures 8 and 9). Owing to the basal/negligible level of VAP-1 and ICAM-1 in TMNK-1 only (Figures 5 and 6) and all HUVECs [34, 35] cultures, these surface molecules plausibly affected the cancer cell adhesion minimally, which explained the minimum effect of the blocking. On the other hand, upregulated level of these surface molecules in the TMNK-1 + 10T1/2|gel culture (Figures 5 and 6) had significant effect on the adhesion of cancer cells (Figure 4), which was revealed by the blocking experiments (Figure 8). In the TMNK-1 + 10T1/2|gel culture, though both cancer cells showed a decreasing tendency under the blocking condition, only MIA-PaCa-2 adhesion decreased significantly (Figure 8). This reflected that liver-microvasculature specific surface molecules of ECs had a critical role which specifically contributed to the higher adhesion of MIA-PaCa-2 than BxPC-3. Notably, higher adhesion of MIA-PaCa-2 than BxPC-3, which was modulated by markers of hLSECs, correlated with the higher propensity of MIA-PaCa-2 than BxPC-3 to form liver metastasis.

Besides the classical role of ICAM-1 on cancer cell adhesion, our studies demonstrated role of VAP-1 on cancer cell adhesion. In addition, our study showed that though the adhesion behavior of a type of cancer cells (e.g., MIA-PaCa-2) can be similar in various EC cultures, the mechanism controlling the adhesion might be different (i.e., effect of VAP-1 and ICAM-1 in the TMNK-1 but not in the HUVECs culture). Therefore, it is crucial to screen various drugs, which target cancer cell adhesion to endothelium, by using* in vitro* cultures which can mimic organ-specific microvasculature property as the culture developed in this study.

After the organ-specific adhesion of cancer cells in the organs, efficacy of the metastasis formation might depend on their migration in the organs [19]. PCs can also involve in the cancer cell migration and colonization in an organ [17, 18]. Therefore, in addition to the cancer cell adhesion, the developed culture would provide an improved culture model for investigating cancer cell migration and colonization (in the gel region).

## 5. Conclusions

We systematically developed a coculture system to mimic liver-specific microvasculature by using continuous cell lines as model cells for organ-specific endothelial cells and pericytes. Endothelial cells in the coculture maintained an elevated level of organ-specific phenotype which was crucial for the correlation between adhesion of cancer cells and their propensity to form liver metastasis* in vivo*. The culture system provides a promising means for screening novel drugs to inhibit cancer cell and endothelium interactions and to investigate details of these interactions in an* in vivo* mimicking organ-specific manner.

## Supplementary Material

Supplementary Figure 1: Images showing continuous monolayer morphology of HUVECs (green) culture on collagen gel surface with (a) or without 10T1/2 (b) on day 4. Scale bar represents 200µm.
