# Supplementary figures and images for: Adhesion of Pancreatic Cancer Cells in a Liver-Microvasculature Mimicking Coculture Correlates with Their Propensity to Form Liver-Specific Metastasis In Vivo
Source: Biomed Res Int. 2014 May 11;2014:241571. doi: 10.1155/2014/241571 (PMC4037581; doi:10.1155/2014/241571)

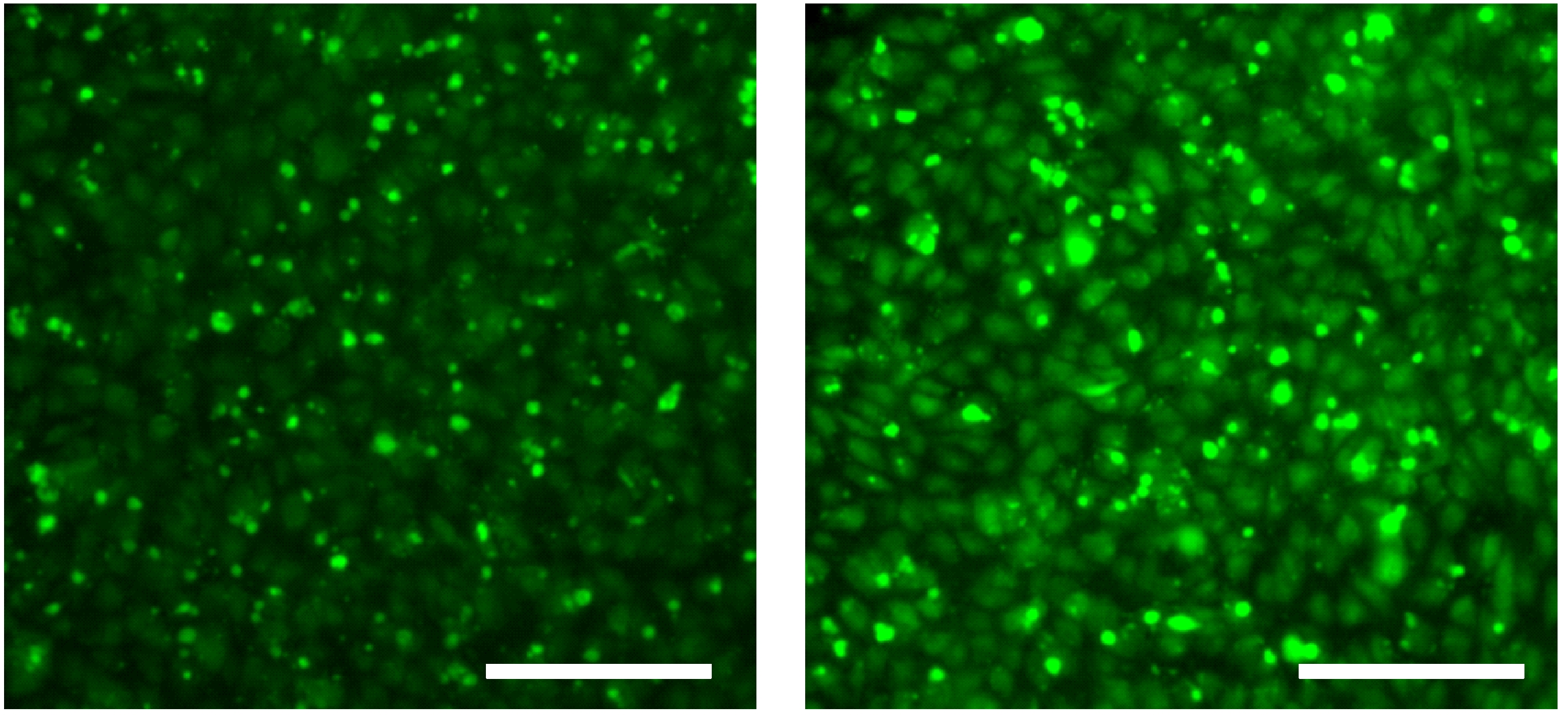

Supplement: Supplementary file 1 — Supplementary Figure 1: Images showing continuous monolayer morphology of HUVECs (green) culture on collagen gel surface with (a) or without 10T1/2 (b) on day 4. Scale bar represents 200µm. [file 241571.f1.jpg]
